# Editorial: Highlights in cultural psychology: language

**DOI:** 10.3389/fpsyg.2025.1703060

**Published:** 2025-09-29

**Authors:** Henrike K. Blumenfeld, Anna B. Cieślicka

**Affiliations:** ^1^Bilingualism & Cognition Laboratory, School of Speech, Language & Hearing Sciences, San Diego State University, San Diego, CA, United States; ^2^Brain and Cognition Laboratory, Department of Psychology and Communication, Texas A&M International University, Laredo, TX, United States

**Keywords:** language learning, language-identity connection, acculturation, dimensions of culture, language-culture relation, language in context, ecolinguistics

A long research tradition has tied language experience and proficiency to cultural identity and competence, from views that language shapes cultural thought, to broader views that language can bias thought patterns and that language and culture can mutually reinforce each other (e.g., Danesi, 2021; Schroeder et al., 2017). Moreover, the process of language learning has been situated in a sociocultural context, with the argument that social networks, environments, and societal attitudes can shape language learning (e.g., Chong et al., 2023).

In the current Research Topic, these themes are pursued and re-examined through fresh quantitative and qualitative lenses that provide wider views of the dynamic systems in which language and culture interact. In four articles, language learning is examined within a broader sociocultural context. Findings show that L2 learners build dynamic bicultural identities (Peng), that cultural identities can shape L2 communication styles (Munkova et al.), and that positive identities and interests in a culture are pre-requisites for the learning and maintenance of language (Gu and Deng; Razmjoo Moghadam and Barani). In *Understanding the cultural identity of EFL learners from the eco-linguistics perspective*, Peng examines Chinese and Western cultural identification characteristics of Chinese college-aged learners of English as a Foreign Language at Bronfenbrenner (1979)'s macro-, meso-, exo-, and micro-levels. In thematic analysis of survey data, aspects of cultural identification are tied to individual experiences. Further, Peng shows that world events can dynamically shift components of cultural identification, for example showing a greater prevalence of Confucian values and national identity in the macro-factor of Chinese cultural identification post-COVID19-pandemic. In *Communication models in a foreign language*, based on the analysis of English communication patterns of university students in Slovakia, Munkova et al. suggest that second language learning occurs within a framework of cultural practices and is influenced by such processes. Specifically, Munkova et al. find that cultural communication norms associated with the mother tongue (e.g., in terms of power distance) transfer into the learners' second language and that communication strategies are co-determined by cognitive style differences (broad-stroke vs. rule-oriented) across participants.

Zooming out to broader policy considerations that often provide the backdrop to language learning, Gu and Deng examine how language and culture planning can be leveraged to enhance national “soft power” in *Rearticulating the promotion of Japanese language and culture*. Based on a decade of available data from the Japan Foundation, the authors argue that the international building of language education programming and language prestige through access to culture is leveraged to support cultural diplomacy. This work is based on the previously identified interconnectedness between language education and language prestige (Baldauf, 2006). As some languages gain prestige through successful international use in language education and beyond, individual speakers often experience prestige differentials across the languages they speak and their associated cultures. Razmjoo Moghadam and Barani further explore these dynamics in *The impact of linguistic vs. cultural imperialism on language learning*. Based on a systematic review of publications in applied linguistics and language teaching, the authors identify and discuss themes related to social and language identities and how they can be impacted by power relations, leading to marginalization of local languages.

Related to the broad theme of language learning, three articles in the current Research Topic shed new light on the types and consequences of language and communication barriers that may be encountered when language and culture do not match the surrounding majorities (Du et al.; Feng and Zhang; Li et al.). In *Cross-cultural nuances in sarcasm comprehension*, Du et al. investigate cross-cultural communication hurdles that may contribute to communication barriers. The authors look at how contrasts in Chinese and American cultural values shape comprehension of sarcasm amongst Hofstede (2011)'s cultural dimensions. The findings show that greater power distance is linked to better sarcasm comprehension across cultures and that collectivism is associated with improved sarcasm comprehension, especially in the Chinese cultural context. These findings highlight that culture drives communication in nuanced ways that show both similarities and differences across contexts.

Feng and Zhang explore psychological adaptation in college students who had come from Macau (where Cantonese is the dominant language) to study in Mainland China (where Mandarin is dominant) in *Stay strong, stay healthy*. While both identity and self-reported proficiency in Mandarin are correlated with students' psychological adaptation in their new environment, identity emerges as the stronger predictor of psychological adaptation. Further, Mandarin proficiency and national Chinese identity are significantly correlated. In a case study of the socioemotional consequences of language barriers in *older* adults, Li et al. analyze data from a national survey in China in *Exploring the relationship between mental health and dialect use*. Analyses show worse self-reported mental health in individuals who speak minority Chinese dialects in regions where Mandarin is the dominant language. Interestingly, this effect is mediated by income inequality and subjective wellbeing, where less income inequality and greater subjective wellbeing reduce the negative impact of minority dialect use on mental health. Thus, both Feng and Zhang and Li et al. document the possibility of worse socioemotional outcomes in the presence of a language mismatch, particularly in the presence of other factors that are also linked to negative outcomes.

Altogether, articles in the current Research Topic further expand our understanding of the deeply entwined relationship between cultural psychology and language, and how this knowledge can be utilized to preserve minority culture's values, beliefs, and customs. The contributions show that, in line with ecological models (Bronfenbrenner, 1979; Chong et al., 2023), language learning and its associated proficiency attainment and socioemotional outcomes can be placed within a broader sociocultural and sociopolitical context (see Figure 1 for an overview). As such, it is important for educators, researchers, and policy makers to acknowledge that language learning and maintenance are impacted by dynamic interactions within complex systems.

**Figure 1:**
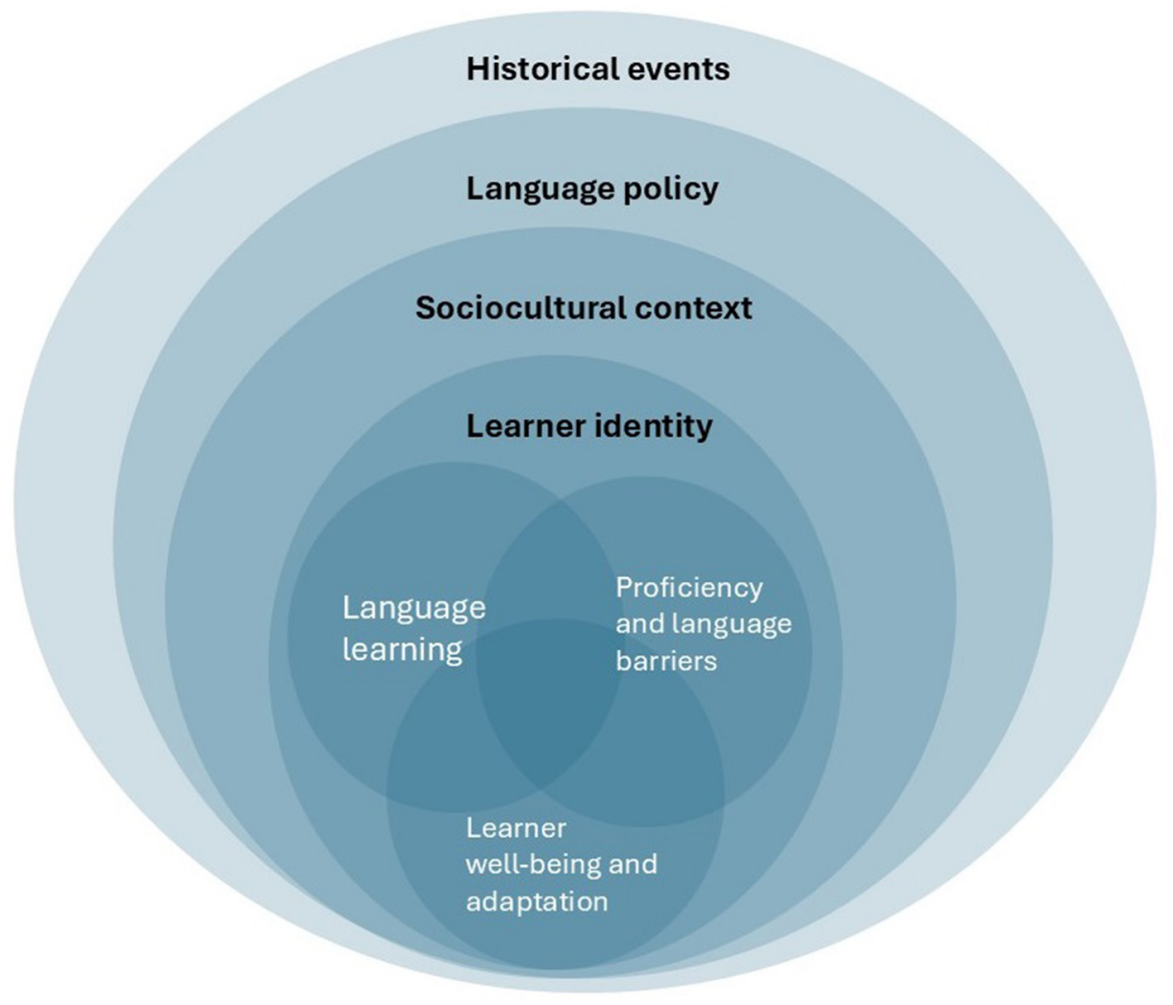
Theoretical landscape, based on previous frameworks by Bronfenbrenner (1979) and Chong et al. (2023), of the articles included in the current special issue. Here, the language learner's cultural identity, as influenced by the sociocultural context (e.g., environmental languages and their prestige, Razmjoo Moghadam and Barani), language policy initiatives (e.g., availability of learning resources, Gu and Deng), and historical events (e.g., the COVID19 pandemic, Peng), is thought to shape their language learning processes, which in turn are tied to their proficiency and encountered language and communication barriers (Du et al.; Munkova et al.), and their wellbeing and ability to adapt to their linguistic and cultural environments (Feng and Zhang; Li et al.).

## References

[B1] Baldauf R. B. Jr. (2006). Rearticulating the case for micro language, planning in a language ecology context. Curr. Issues Lang. Plan. 7, 147–170. 10.2167/cilp092.036373267

[B2] Bronfenbrenner U. (1979). The Ecology of Human Development: Experiments by Nature and Design. Cambridge, MA; London: Harvard University Press. 10.4159/9780674028845

[B3] Chong S. W. Isaacs T. McKinley J. (2023). Ecological systems theory and second language research. Lang. Teach. 56, 333–348. 10.1017/S0261444822000283

[B4] Danesi M. (2021). Linguistic Relativity Today: Language, Mind, Society, and the Foundations of Linguistic Anthropology, 1st Edn. New York, NY: Routledge. 10.4324/9781003001669

[B5] Hofstede G. (2011). Dimensionalizing cultures: the Hofstede model in context. Psychol. Cult. 2:1014. 10.9707/2307-0919.1014

[B6] Schroeder S. R. Lam T. Q. Marian V. (2017). Linguistic predictors of cultural identification in bilinguals. Appl. Ling. 38, 463–488. 10.1093/applin/amv049 PMC5603315 28936014

